# Cellular Prion Protein Is Essential for Myocardial Regeneration but Not the Recovery of Left Ventricular Function from Apical Ballooning

**DOI:** 10.3390/biomedicines10010167

**Published:** 2022-01-13

**Authors:** Jiunn-Jye Sheu, Han-Tan Chai, John Y. Chiang, Pei-Hsun Sung, Yi-Ling Chen, Hon-Kan Yip

**Affiliations:** 1 Division of Thoracic and Cardiovascular Surgery, Department of Surgery, Kaohsiung Chang Gung Memorial Hospital, College of Medicine, Chang Gung University, Kaohsiung 83301, Taiwan; cvsjjs@gmail.com; 2 Institute for Translational Research in Biomedicine, Kaohsiung Chang Gung Memorial Hospital, Kaohsiung 83301, Taiwan; e12281@cgmh.org.tw (P.-H.S.); rylchen.msu@gmail.com (Y.-L.C.); 3 Center for Shockwave Medicine and Tissue Engineering, Kaohsiung Chang Gung Memorial Hospital, Kaohsiung 83301, Taiwan; 4 Division of Cardiology, Department of Internal Medicine, Kaohsiung Chang Gung Memorial Hospital, College of Medicine, Chang Gung University, Kaohsiung 83301, Taiwan; chaiht@mail.cgmh.org.tw; 5 Department of Computer Science and Engineering, National Sun Yat-sen University, Kaohsiung 804201, Taiwan; chiang@cse.nsysu.edu.tw; 6 Department of Healthcare Administration and Medical Informatics, Kaohsiung Medical University, Kaohsiung 80708, Taiwan; 7 Department of Medical Research, China Medical University Hospital, China Medical University, Taichung 40402, Taiwan; 8 Department of Nursing, Asia University, Taichung 41354, Taiwan; 9 Division of Cardiology, Department of Internal Medicine, Xiamen Chang Gung Hospital, Xiamen 361028, China

**Keywords:** takotsubo cardiomyopathy, cellular prion protein, Stelazine, cell stress

## Abstract

This study tested the hypothesis that cellular prion protein (PrP^C^) played an essential role in myocardial regeneration and recovery of left ventricular ejection fraction (LVEF) from apical takotsubo cardiomyopathy (TCM) induced by transaortic constriction (TAC). In vitro study was categorized into G1 (H9C2), G2 (H9C2-overexpression-PrP^C^), G3 (H9C2-overexpression-PrP^C^ + Stelazine/1 uM), and G4 (H9C2 + siRNA-PrP^C^), respectively. The results showed that the protein expressions of PrP^C^, cell-stress signaling (p-PI3K/p-Akt/p-m-TOR) and signal transduction pathway for cell proliferation/division (RAS/c-RAF/p-MEK/p-ERK1/2) were lowest in G1, highest in G2, significantly higher in G3 than in G4 (all *p* < 0.001). Adult-male B6 mice (n = 30) were equally categorized in group 1 (sham-control), group 2 (TAC) for 14 days, then relieved the knot and administered BrdU (50 ug/kg/intravenously/q.6.h for two times from day-14 after TAC) and group 3 (TAC + Stelazine/20 mg/kg/day since day 7 after TAC up to day 21 + BrdU administered as group 2), and animals were euthanized at day 28. The results showed that by day 28, the LVEF was significantly higher in group 1 than in groups 2/3 and significantly higher in group 3 than in group 2, whereas the LV chamber size exhibited an opposite pattern of LVEF (all *p* < 0.0001). The protein expressions of PrP^C^/p-PI3K/p-Akt/p-m-TOR/cyclin D/cyclin E and cellular-proliferation biomarkers (Ki67/PCNA/BrdU) exhibited an opposite pattern of LVEF (all *p* < 0.0001) among the three groups, whereas the protein expressions of RAS/c-RAF/p-MEK/p-ERK1/2 were significantly and progressively increased from groups 1 to 3 (all *p* < 0.0001). In conclusion, PrP^C^ participated in regulating the intrinsic response of cell-stress signaling and myocardial regeneration but did not offer significant benefit on recovery of the heart function in the setting of TCM.

## 1. Introduction

Apical ballooning syndrome, also known as takotsubo cardiomyopathy (TCM), apical ballooning cardiomyopathy, stress-induced cardiomyopathy, or simply stress cardiomyopathy, is a type of non-ischemic cardiomyopathy in which there is a sudden temporary weakening of the myocardium [1,2,3]. TCM is an acute cardiac syndrome characterized by transient regional left ventricular (LV) systolic dysfunction in the absence of obstructive coronary artery disease [4]. Systolic heart failure (HF) is the most common complication and merits early detection and treatment [5].

Studies report that 1.7–2.2% of patients originally treated as suspected acute coronary events were subsequently diagnosed with apical ballooning syndrome. In Japanese, “takotsubo” means “fishing pot for trapping octopus,” and the left ventricle of a patient diagnosed with this condition indeed resembles that shape [6,7].

It is noted that cardiac markers, specifically troponin I and T, are elevated in more than 90% of patients with apical ballooning syndrome, although to a lesser magnitude than in ST-segment elevation myocardial infarction (STEMI). The brain natriuretic peptide level is also frequently elevated [8] due to heart failure/pressure overload.

Treatment options for TCM are largely conservative, and medical treatment, such as beta-blockers [4], at least in the short term, aims to counter the ensuing systolic dysfunction. Serial imaging studies, including transthoracic echocardiography, are necessary to ensure the resolution of cardiomyopathy. Patients who may be found to have LV thrombus, which occurs in 5% of patients with TCM, require anticoagulation. Thereafter, annual clinical follow-up is advised because the long-term outcomes and natural history of TCM in some patients are unknown [9].

The prognostic outcome in TCM is viewed to be quite suitable because as estimated, nearly 95% of patients experience complete recovery within a few months. The recurrence rate varies but is estimated at about 3%. Estimates of mortality rates have been found to be at a range from 1% to 3.2% [10]. Complications occur in 20% of TCM cases and include mechanical and electrical events as the following: LV failure with or without pulmonary edema, cardiogenic shock, LV outflow obstruction, mitral regurgitation, ventricular arrhythmias, LV mural thrombus formation, or free-wall rupture.

Surprisingly, despite the underlying mechanisms initiated, this disease entity has been keenly investigated, including (1) adrenergic overstimulation (i.e., acute hyperactivity of the local sympathetic system), (2) epicardial coronary/microvascular spasm, (3) stress provocation, or (4) catecholamine-induced myocardial stunning, the exact underlying mechanisms remain poorly understood. Not only the aforementioned issues, i.e., 1 to 4 main points, but recent studies have further clarified that an alteration of sympathetic tone with autonomic dysfunction and cardiac denervation [11] played an essential role in the development of TCM. Intriguingly, an altered distribution of beta-receptors has been precisely identified in the cardiac apex of TCM patients by cardiac metaiodobenzylguanidine (MIBG) scintigraphy [11,12] that added to over-sympathetic drive and could, therefore, result in typically apical ballooning [11,12]. Studies have even more proved that this alteration was persistent for the long term [13].

More surprisingly, the underlying mechanisms involved in myocardial regeneration and recovery of LV function have not yet been investigated, highlighting that the comprehension of pathophysiological pathways of anabiosis (i.e., heart functional recovery) from TCM is still an unmet need.

The PrP^C^ is a cell-surface glycosylphosphatidylinositol-anchored glycoprotein that has been identified to attach to the plasma membrane [14]. The PrP^C^ has been exhibited to play a crucial role in various cellular functions, including the cell cycle, cell growth, cell proliferation, cell–cell adhesion, cell migration, and the maintenance of cell shape [15,16]. PrP^C^ is strongly expressed in the central nervous system (CNS) and can act as a regulator of neuronal development, differentiation, and neurite outgrowth [17]. Interestingly, some studies have previously displayed that PrP^C^ regulates self-renewal, differentiation, and functional enhancement in stem/progenitor cells [18,19,20]. Later, some growing data indicate that the PrP^C^ is involved in cancer cell proliferation, cell survival, resistance to apoptosis, and metastasis [21,22,23]. In addition, PrP^C^ has been established to play an extremely critical role in the invasive capacity and the acquisition of multidrug resistance in cancer cells [24]. Interestingly, a recent study has keenly investigated hyperglycemia attenuated anticancer effects and augmented its cardiotoxicity in cellular models through mechanisms of regulating NLRP3 inflammasome and MyD88 signaling [25], suggesting hyperglycemia increased cytokine storm in human cancer cells and cardiotoxic induced by anticancer therapies [25]. These findings may raise consideration of the correlation between PrPC and NLRP3 inflammasome and its role in cardiac dysfunctions induced by TCM. However, whether PrP^C^ plays an important role in the recovery of TCM is still unclear. Using in vitro and in vivo studies, we tested the hypothesis that PrP^C^ may participate in the regeneration of damaged myocardium and recover LV function in the setting of TCM.

## 2. Materials and Methods

### 2.1. Ethics

All animal procedures were approved by the Institute of Animal Care and Use Committee at Kaohsiung Chang Gung Memorial Hospital (Affidavit of Approval of Animal Use Protocol No. 2018122204) and performed in accordance with the Guide for the Care and Use of Laboratory Animals. Animals were housed in an Association for Assessment and Accreditation of Laboratory Animal Care International (AAALAC; Frederick, MD, USA)-approved animal facility in our hospital with controlled temperature and light cycles (24 °C and 12/12 light cycle).

### 2.2. Animal Model of Takotsubo Cardiomyopathy (TCM) (i.e., Apical Ballooning)

A development of apical ballooning in animal models by using transverse aortic constriction (TAC) has been performed by William M. Chilian [26]. Additionally, we have previously created a permanent TAC animal model for inducing hypertrophic cardiomyopathy, heart failure, and pulmonary hypertension for individual study, respectively [27,28,29]. Accordingly, for the purpose of the present study, we could create a TCM animal model by following the guideline outlined.

### 2.3. Procedure of TAC for Induction of TCM

The pathogen-free, adult-male mice (n = 30) weighing 250 g (Charles River Technology, BioLASCO, Taiwan) were used for the present study. In detail, all animals were placed in a supine position under anesthesia with 2.0% inhalational isoflurane on a warming pad at 37 °C and then intubated with positive-pressure ventilation (180 mL/min) with room air using a small animal ventilator (SAR-830/A, CWE, Inc.,Scotts Valley, CA, USA) for the TAC procedure. Under sterile conditions, the heart was exposed via a left thoracotomy procedure. TAC was induced by tying firmly but with a slipknot (i.e., a knot that could be undone by a pull) in the ascending aorta with a piece of 7-0 prolene on a 27# needle between the aortic arch (i.e., just beyond the right common carotid artery (CCA)) and left CCA. The needle was then removed, leaving a constricted aorta. These animals were then assigned to groups 2 and 3, respectively. Only a left thoracotomy without TAC was performed for group 1 animals. After the TAC procedure, the thoracotomy wound was closed, and the animals were allowed to recover from anesthesia in a portable animal intensive care unit (ThermoCare^®^) for 24 h.

By day 14, after the TAC procedure, opening the chest wall again was performed, and the slipknot was removed from the animals in groups 2 and 3. The heart function of animals in each group was serially assessed during the study period. The study animals were euthanized on day 28 after TAC induction.

### 2.4. Animal Grouping and Treatment Strategy

The animals were categorized into group 1 (sham-control), group 2 (TAC for 14 days, then relieved the knot and administered BrdU (50 ug/kg q.6.h for two times) from day 14 after TAC by intravenous administration) and group 3 (TAC + Stelazine (20 mg/kg per day orally) since day 7 after TAC up to day 21 + BrdU administered as group 2). Animals were euthanized on day 28, and the heart in each animal was harvested for individual study. Therefore, groups 1, 2, and 3 were referred to SC, TCM, and TCM-Stelazine, respectively.

Stelazine is an analog of phenothiazine, which inhibits the protein expression of cellular prion protein (PrP^C^) and has the capacity of α-adrenergic blockage. Additionally, Bromodeoxyuridine (5-bromo-2′-deoxyuridine, BrdU) is a synthetic nucleoside that is an analog of thymidine. BrdU is commonly used in the detection of proliferating cells in living tissues. 5-Bromodeoxycytidine is deaminated to form BrdU, which was used in the present study for the purpose of detecting cell proliferation. The dosages of BrdU and Stelazine to be used in the present study were based on our [30] and previous [31] studies.

### 2.5. LV Functional Assessment by Echocardiography

The procedure and protocol were based on our previous report [32]. In detail, transthoracic echocardiography (Vevo 2100, Visualsonics, Toronto, ON, Canada) was serially performed in animals from each group prior and after TAC induction at the time points of days 7, 14, 21, and 28 by a cardiologist blinded to the experimental design. M-**mode** standard **two-dimensional** (2D) left parasternal **long-axis** echocardiographic examination was conducted. Left ventricular internal dimensions (i.e., left ventricular end-systolic diameter (LVESd) and left ventricular end-diastolic diameter (LVEDd)) were measured at mitral valve level of the left ventricle, as per the American Society of Echocardiography (Morrisville, NC, USA) leading-edge methodology, using at least three consecutive cardiac cycles. Left ventricular ejection fraction (LVEF) was calculated as follows: LVEF (%) = [(LVEDd^3^−LVESd^3^)/LVEDd^3^] × 100%.

### 2.6. The Procedure and Protocol for PrP^c^ Overexpression and Silencing

The procedure and protocol have been reported by our recent study [33]. The pCS6-PRNP plasmid was purchased from Transomic Technologies (Huntsville, AL, USA). The plasmid transfection process was carried out with Lipofectamine 3000 according to the manual steps. The steps were briefly described as follows: 10 μg plasmid and 20 μL Lipofectamine 3000 were first incubated at room temperature for 15 min, followed by overnight incubation of cells and Lipofectamine (i.e., mixed them together), and related experiments were carried out.

For the PrP^c^ silencing procedure, 30 pmol siRNA (targeting sequence is 5′-GCCCUCUUUGUGACUACAU-3′) was incubated with 50 μL Lipofectamine RNAiMAX for 15 min at room temperature, followed by overnight incubation of cells and Lipofectamine (i.e., mixed them together), and related experiments were carried out.

### 2.7. The Procedure and Protocol for H9C2 Cell Culturing and Treated by Hydrogen Peroxide (H_2_O_2_), Isoproterenol, Stelazine, or PrP^c^ Overexpression

Briefly, 1 × 10^6^ cells were first seeded in a 10 cm dish. After 24 h cell culture, the cells were treated with Stelazine (1 mM) for 24 h, followed by PrP^c^ overexpression or silencing for 24 h. Finally, the cells were collected for Western blot analysis.

Additionally, 3 × 10^6^ cells were seeded in a 10 cm dish. After 24 h of cell culture, the H9C2 cells were treated with H_2_O_2_ (100 mM) or isoproterenol (50 mM). After 4 h of incubation, cell extracts were collected for Western blotting.

### 2.8. Immunohistochemical (IHC) and Immunofluorescent (IF) Staining

The procedure and protocol for IHC and IF staining have been described in our previous reports [27,28,29,30]. For IHC and IF staining, rehydrated paraffin sections were first treated with 3% H_2_O_2_ and incubated with Immuno-Block reagent (BioSB, Santa Barbara, CA, USA) for 30 min at room temperature. Sections were then incubated with primary antibodies specifically against proliferating cell nuclear antigen (PCNA) (1:2000, Cell Signaling Technology, Danvers, MA, USA), BrdU (1:10, BD Pharmingen™ BrdU In-Situ Detection Kit), and Ki67 (1:500, Abcam, Trumpington, Cambridge, UK), while sections incubated with the use of irrelevant antibodies served as controls. Three sections of LV myocardial specimen from each mouse were analyzed. For quantification, three randomly chosen HPFs (200× or 400× for IHC and IF studies) were analyzed in each section. The mean number of positively stained cells per HPF for each animal was then determined by summation of all numbers divided by 9.

### 2.9. Western Blot Analysis

The procedure and protocol for Western blot analysis have been described in our previous reports [27,28,29,30]. Briefly, equal amounts (50 μg) of protein extracts were loaded and separated by SDS-PAGE using acrylamide gradients. After electrophoresis, the separated proteins were transferred electrophoretically to a polyvinylidene difluoride (PVDF) membrane (GE Healthcare, Buckinghamshire, UK). Nonspecific sites were blocked by incubation of the membrane in blocking buffer (5% nonfat dry milk in T-TBS (TBS containing 0.05% Tween 20) overnight. The membranes were incubated with the indicated primary antibodies (PrPC (1:1000, Abcam), phosphorylated (p)-PI3K (1:1000, Cell Signaling Technology, Danvers, MA, USA), total PI3K (1:1000, Cell Signaling Technology, Danvers, MA, USA), p-Akt (1:1000, Cell Signaling Technology, Danvers, MA, USA), total Akt (1:1000, Cell Signaling Technology, Danvers, MA, USA), p-m-TOR (1:1000, Cell Signaling Technology, Danvers, MA, USA), total m-TOR (1:1000, Cell Signaling Technology, Danvers, MA, USA), RAS (1:1000, Abcam), c-RAF (1:1000, Abcam), p-MEK (1:1000, Cell Signaling Technology, Danvers, MA, USA), p-ERK1/2 (1:3000,Millipore, Burlington, MA, USA, Danvers, MA, USA), PTEN (1:1000, Abcam), cyclin D (1:1000, Abcam), cyclin E (1:1000, Abcam), cleaved caspase 3 (1:1000, Cell Signaling Technology, Danvers, MA, USA), cleaved Poly (ADP-ribose) polymerase (PARP) (1:1000, Cell Signaling Technology, Danvers, MA, USA), transforming growth factor (TGF)-ß (1:1000, Abcam) and Smad3 (1:1000, Cell Signaling Technology, Danvers, MA, USA), brain natriuretic peptide (BNP) (1:1000, Abcam), γ-H2AX (1:1000, Cell Signaling Technology, Danvers, MA, USA), alpha myelin heavy chain (α-MHC) (1:1000, Santa Cruz, Dallas, TX, USA) and ß-MHC (1:1000, Santa Cruz)) for 1 h at room temperature. Horseradish peroxidase-conjugated anti-rabbit immunoglobulin IgG (1:2000, Cell Signaling Technology, Danvers, MA, USA), Danvers, MA, USA) was used as a secondary antibody for one-hour incubation at room temperature. The washing procedure was repeated eight times within one hour. Immunoreactive bands were visualized by enhanced chemiluminescence (ECL; Amersham Biosciences, Amersham, UK) and exposed to Biomax L film (Kodak, Rochester, NY, USA). For quantification, ECL signals were digitized using Labwork software (UVP, Waltham, MA, USA).

### 2.10. Histological Quantification of Myocardial Fibrosis

The procedure and protocol have been described in detail in our previous reports [28,29]. Briefly, hematoxylin and eosin (H&E) and Masson’s trichrome staining were used to identify the infarct area and fibrosis of LV myocardium, respectively. Three serial sections of LV myocardium in each animal were prepared at 4 µm thickness by Cryostat (Leica CM3050S, Buffalo Grove, IL, USA). The integrated area (µm^2^) of infarct area and fibrosis on each section was calculated using the Image Tool 3 (IT3) image analysis software (University of Texas, Health Science Center, San Antonio, UTHSCSA; Image Tool for Windows, Version 3.0, USA). Three randomly selected high-power fields (HPFs) (100×) were analyzed in each section. After determining the number of pixels in each infarct and fibrotic area per HPF, the numbers of pixels obtained from three HPFs were calculated. The procedure was repeated in two other sections for each animal. The mean pixel number per HPF for each animal was then determined by calculating all pixel numbers and dividing by 9. The mean integrated area (µm^2^) of fibrosis in LV myocardium per HPF was obtained using a conversion factor of 19.24 (since 1 µm^2^ corresponds to 19.24 pixels). This method was also applied for the identification of collagen deposition in the myocardium.

### 2.11. Statistical Analysis

Quantitative data were expressed as mean ± SD. Statistical analysis was adequately performed by ANOVA followed by Bonferroni multiple comparison procedure. Statistical analysis was performed using SAS statistical software for Windows version 13 (SAS Institute, Cary, NC, USA). A probability value < 0.05 was considered statistically significant.

## 3. Results

### 3.1. Isoproterenol and H_2_O_2_ Suppressed the PrP^C^ and Cell-Stress Signaling Pathway in H9C2 Cells

**Figure 1:**
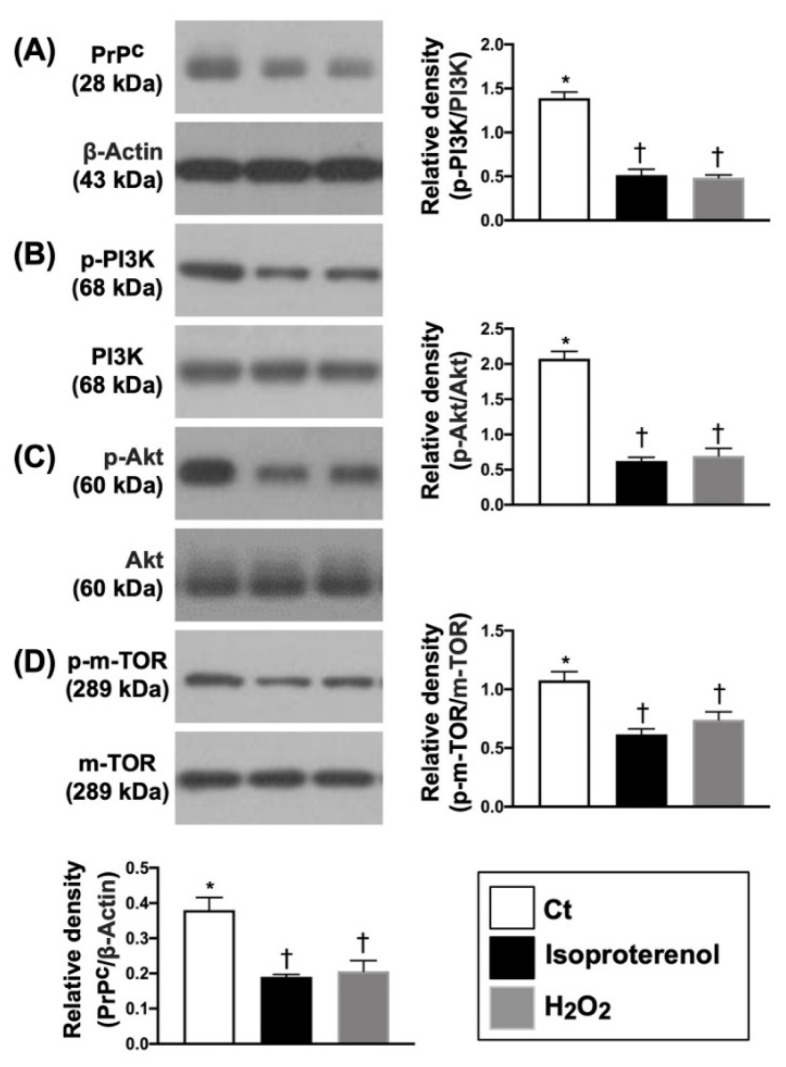
Isoproterenol and H_2_O_2_ suppressed the PrP^C^ and cell-stress signaling pathway in H9C2 cells. (**A**) Protein expression of cellular prion protein (PrP^C^), * vs. †, *p* < 0.01. (**B**) Protein expression of phosphorylated (p)-PI3K, * vs. †, *p* < 0.001. (**C**) Protein expression of p-Akt, * vs. †, *p* < 0.0001. (**D**) Protein expression of p-m-TOR, * vs. †, *p* < 0.001. n = 3 in each group. H_2_O_2_ = hydrogen peroxide; PI3K = Phosphoinositide 3-kinase; m-TOR = mammalian target of rapamycin (m-TOR); RAC(Rho family)-alpha serine/threonine-protein kinase.

To investigate the impact of isoproterenol and H_2_O_2_ on the protein level of PrP^C^ and cell-stress signaling biomarkers, Western blot analysis was used. The in vitro study demonstrated that the protein expressions of PrP^C^ and p-PI3K/p-Akt/p-m-TOR in H9C2 treated by isoproterenol (50 uM) and H_2_O_2_ (100 uM) were significantly suppressed as compared with the control group (i.e., H9C2 cells only), suggesting that the intrinsic responses of cell proliferation and cell-stress signaling were suppressed by β adrenoceptor agonist and oxidative stress (Figure 1).

### 3.2. PrP^C^-Regulated the Cell-Stress and Cell-Proliferation/Survival Signaling Pathways in H9C2 Cells

**Figure 2:**
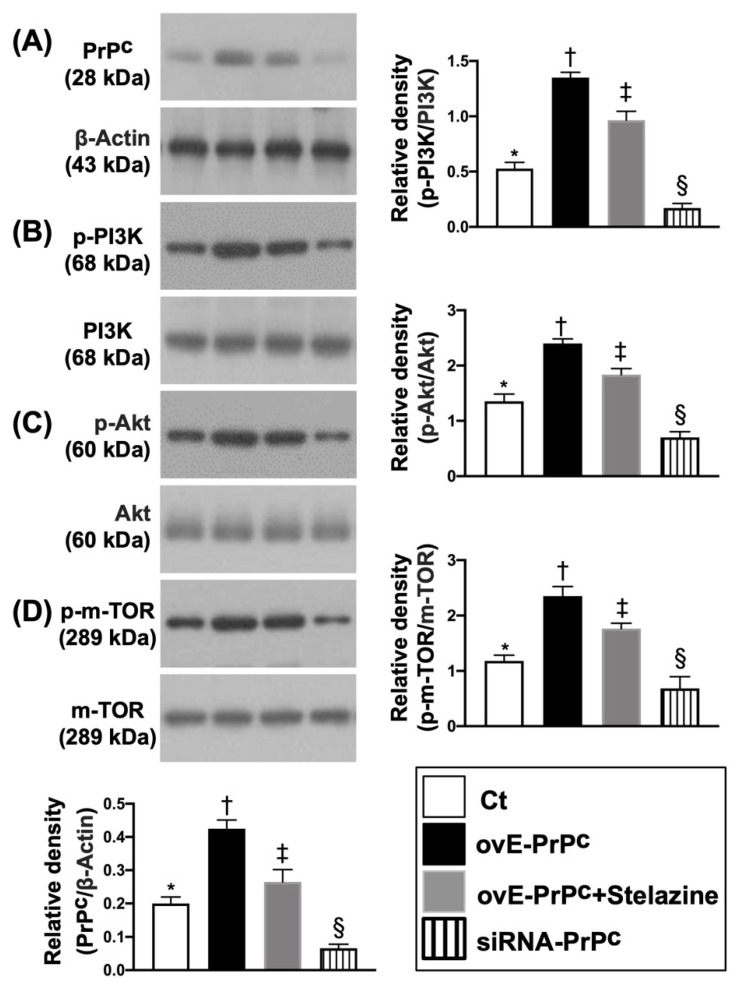
PrP^C^ regulated the cell-stress signaling pathway in H9C2 cells. (**A**) Protein expression of PrP^C^, * vs. other groups with different symbols (†, ‡, §), *p* < 0.0001. (**B**) Protein expression of phosphorylated (p)-PI3K, * vs. other groups with different symbols (†, ‡, §), *p* < 0.0001. (**C**) Protein expression of p-Akt, * vs. other groups with different symbols (†, ‡, §), *p* < 0.0001. (**D**) Protein expression of p-m-TOR), * vs. other groups with different symbols (†, ‡, §), *p* < 0.0001. All statistical analyses were performed by one-way ANOVA, followed by Bonferroni multiple comparison post-hoc test (n = 3 for each group). Symbols (*, †, ‡, §) indicate significance (at 0.05 level). Ct = control group (i.e., H9C2 cells only); ovE-PrP^C^ = overexpression of PrP^C^ in H9C2 cells; siRNA-PrP^C^ = siRNA knockdown PrP^C^ in H9C2 cells; PI3K = Phosphoinositide 3-kinase; m-TOR = mammalian target of rapamycin (m-TOR); RAC(Rho family)-alpha serine/threonine-protein kinase.

**Figure 3:**
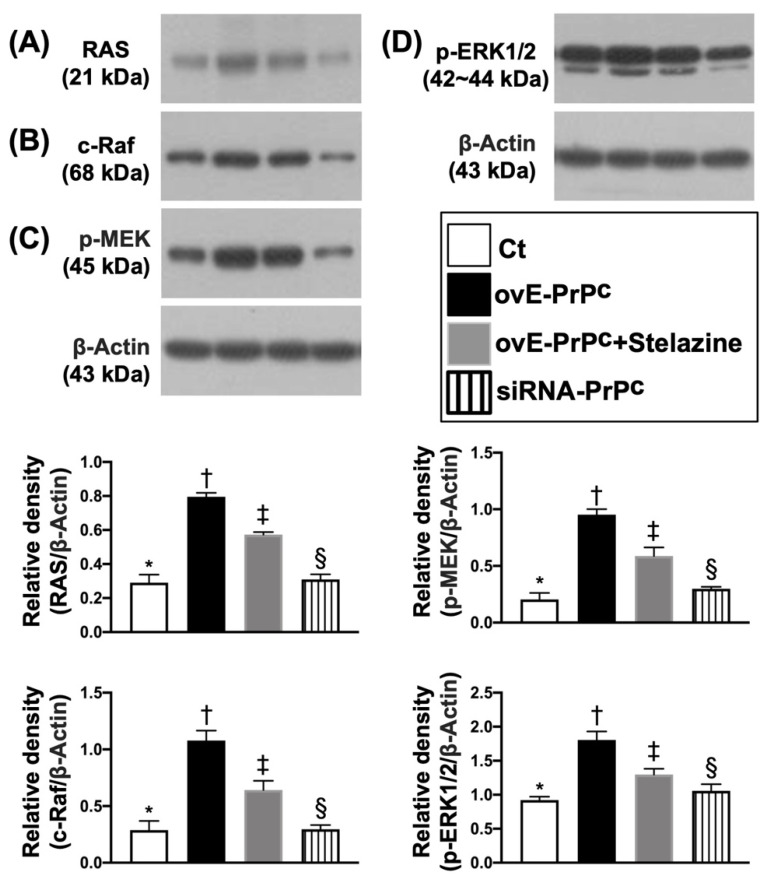
PrP^C^-regulated epidermal growth factor (EGF) signaling pathway in H9C2 cells. (**A**) Protein expression of RAS, * vs. other groups with different symbols (†, ‡, §), *p* < 0.0001. (**B**) Protein expression of cellular (c)-Raf, * vs. other groups with different symbols (†, ‡, §), *p* < 0.0001. (**C**) Protein expression of phosphorylated (p)-MEK, * vs. other groups with different symbols (†, ‡, §), *p* < 0.0001. (**D**) Protein expression of p-ERK)1/2, * vs. other groups with different symbols (†, ‡, §), *p* < 0.0001. All statistical analyses were performed by one-way ANOVA, followed by Bonferroni multiple comparison post-hoc test (n = 3 for each group). Symbols (*, †, ‡, §) indicate significance (at 0.05 level). Ct = control group (i.e., H9C2 cells only); ovE-PrP^C^ = overexpression of PrP^C^ in H9C2 cells; siRNA-PrP^C^ = siRNA knockdown PrP^C^ in H9C2 cells; Ras = Rat sarcoma; ERK = extracellular signal-regulated protein kinase; MEK = mitogen-activated protein kinase kinase; Raf = proto-oncogene c-RAF.

To elucidate whether the PrP^C^-regulated cell-stress signaling pathway (i.e., p-PI3K/p-Akt/p-m-TOR), the conditions of G1 (H9C2 cells only), G2 (overexpression of PrP^C^ in H9C2 cells), G3 (overexpression of PrPC in H9C2 cells + Stelazine (1 uM), i.e., an inhibitor of PrPC) and G4 (siRNA knockdown PrP^C^ in H9C2 cells) were used in the in vitro study. The results showed that the protein expression of PrP^C^ was highest in G2, lowest in G4, and significantly higher in G3 than G1. Additionally, the protein expressions of p-PI3K, p-Akt, and p-m-TOR exhibited an identical pattern of PrP^C^ among the four groups (Figure 2). Furthermore, the signal transduction pathway in biology for control cell growth and cell death (i.e., RAS, c-RAF, p-MEK1/2, p-ERK1/2) also displayed an identical pattern of PrP^C^ among the four groups, implicating that the PrP^C^ regulated these two signaling pathways in situations of cell stress and division (Figure 3).

### 3.3. Time Courses of LVEF in B6 Mice

**Figure 4:**
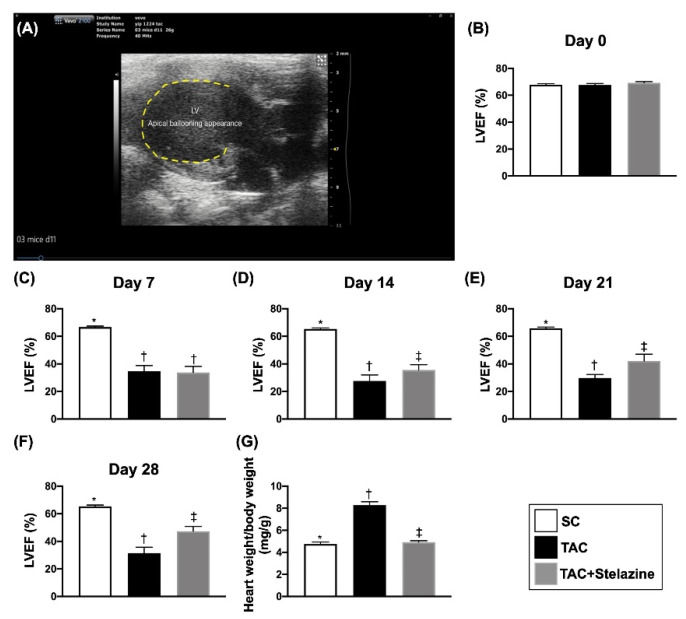
Illustrating the picture of apical ballooning, time courses of LVEF in B6 mice prior to and after TAC procedure, and day 28’s ratio of heart weight to body weight. (**A**) Illustrating the transaortic constriction (TAC) procedure successfully induced morphological feature of takotsubo cardiomyopathy (TCM) (i.e., apical ballooning) by day 14 after TAC procedure. The yellow dotted line indicated an apical aneurysm-like dilatation, mimicking TCM in an animal. (**B**) LVEF at day 0, *p* > 0.5. (**C**) LVEF at day 7 after TCA, * vs. †, *p* < 0.0001. (**D**) LVEF at day 15 after TCA, * vs. other groups with different symbols (†, ‡), *p* < 0.0001. (**E**) LVEF at day 21 after TAC, * vs. other groups with different symbols (†, ‡), *p* < 0.0001. (F) LVEF at day 28 after TAC, * vs. other groups with different symbols (†, ‡), *p* < 0.0001. (**G**) The ratio of heart weight to body weight by day 28 after the whole heart to be harvested. * vs. other groups with different symbols (†, ‡), *p* < 0.0001. All statistical analyses were performed by one-way ANOVA, followed by Bonferroni multiple comparison post-hoc test (n = 10 for each group). Symbols (*, †, ‡) indicate significance (at 0.05 level). SC = sham-operated control TAC = transverse aortic constriction; LVEF = left ventricular ejection fraction.

At baseline prior to the transaortic constriction (TAC) procedure, the LVEF did not differ among the three groups. However, by day 7 after the TAC procedure, the LVEF was significantly higher in group 1 (i.e., sham-operated control) than in group 2 (i.e., TAC only) and group 3 (i.e., TAC + Stelazine), but it showed no difference between groups 2 and 3. On the other hand, by days 15, 21, and 28 after the TAC procedure, this parameter was significantly higher in group 1 than in groups 2 and 3 and significantly higher in group 3 than in group 2, suggesting that it could be attributed to a reduction in afterload resulted from Stelazine therapy (Figure 4).

### 3.4. Protein Expressions of PrP^C^, Cell Stress and Proliferation Signalings by Day 28 after TAC Procedure 

**Figure 5:**
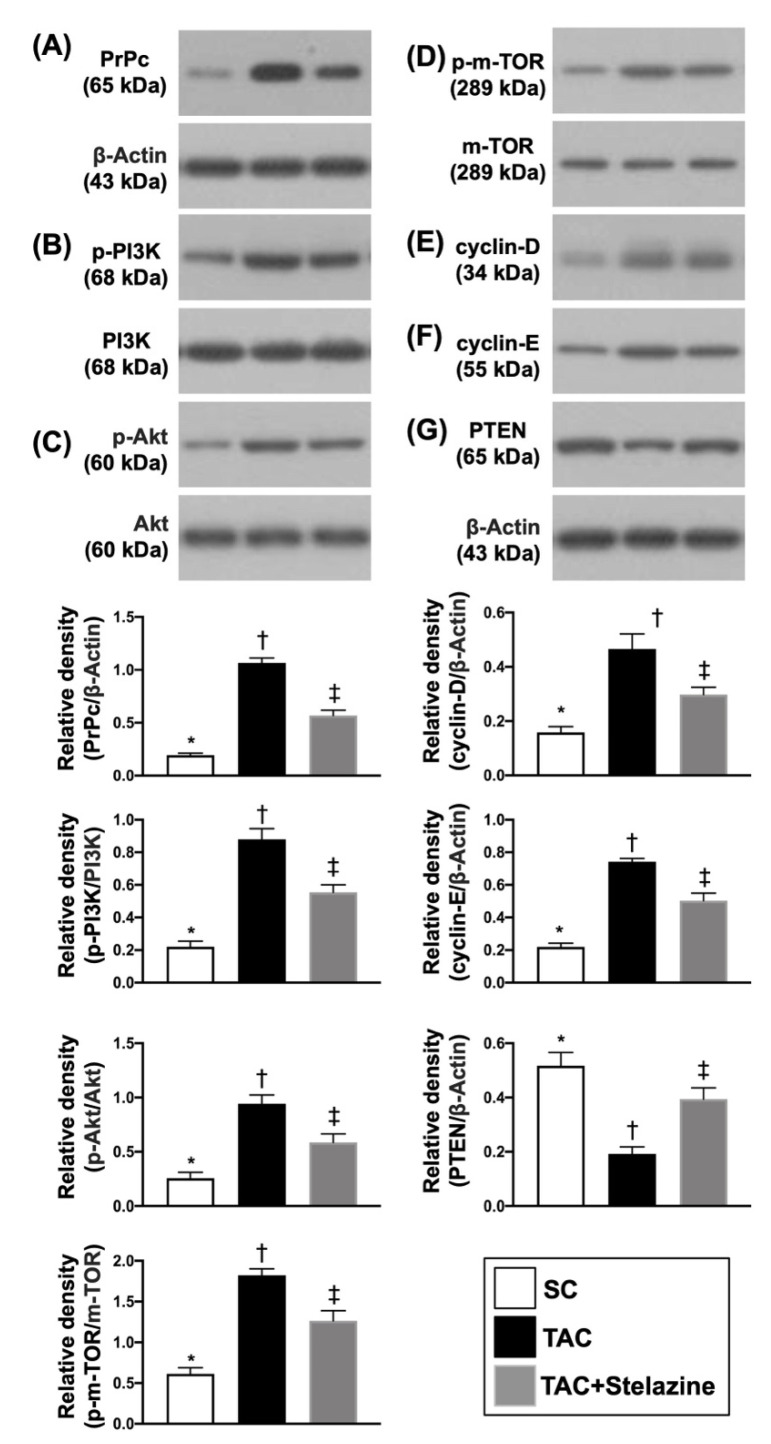
Protein expressions of PrP^C^, cell stress, and proliferation signalings by day 28 after TAC procedure. (**A**) Protein expression of PrP^C^, * vs. other groups with different symbols (†, ‡), *p* < 0.0001. (**B**) Protein expression of p-PI3K, * vs. other groups with different symbols (†, ‡), *p* < 0.0001. (**C**) Protein expression of p-AKT, * vs. other groups with different symbols (†, ‡), *p* < 0.0001. (**D**) Protein expression of p-m-TOR, * vs. other groups with different symbols (†, ‡), *p* < 0.0001. (**E**) Protein expression of cyclin D, * vs. other groups with different symbols (†, ‡), *p* < 0.0001. (**F**) Protein expression of cyclin E, * vs. other groups with different symbols (†, ‡), *p* < 0.0001. (**G**) Protein expression of P-TEN, * vs. other groups with different symbols (†, ‡), *p* < 0.0001. All statistical analyses were performed by one-way ANOVA, followed by Bonferroni multiple comparison post-hoc test (n = 6 for each group). Symbols (*, †, ‡) indicate significance (at 0.05 level). SC = sham-operated control TAC = transverse aortic constriction; PI3K = phosphoinositide 3-kinase; m-TOR = mammalian target of rapamycin (m-TOR); RAC(Rho family)-alpha serine/threonine-protein kinase; P-TEN = phosphatase and tensin homolog.

To elucidate whether the protein levels of PrP^C^ and cell-stress signaling would be upregulated by TCM induced by TAC, the Western blot was performed for the harvested LV specimen. As we expected, the protein expression of PrP^C^ and the protein expressions of p-PI3K, p-AKT, and p-m-TOR, three indicators of cell-stress signaling, were significantly increased in group 2 than in groups 1 and 3, and significantly increased in group 3 than in group 1. Additionally, the protein expressions of cyclin D and cyclin E, two contributors for progression through the G1 phase of the cell cycle to induce cell migration (i.e., cell cycle G1/S transition), displayed an identical pattern of PrP^C^ among the three groups, suggesting that TCM elicited the generation of PrP^C^ in LV myocardium, which in turn regulated the productions of cell stress and cell proliferation/cell cycle proteins. On the other hand, the protein expression of phosphatase and tensin homolog (P-TEN), a tumor suppression biomarker, displayed an opposite pattern of PrP^C^ among the three groups (Figure 5).

### 3.5. Protein Expressions of Cell-Proliferation/Survival Signaling by Day 28 after TAC Procedure

**Figure 6:**
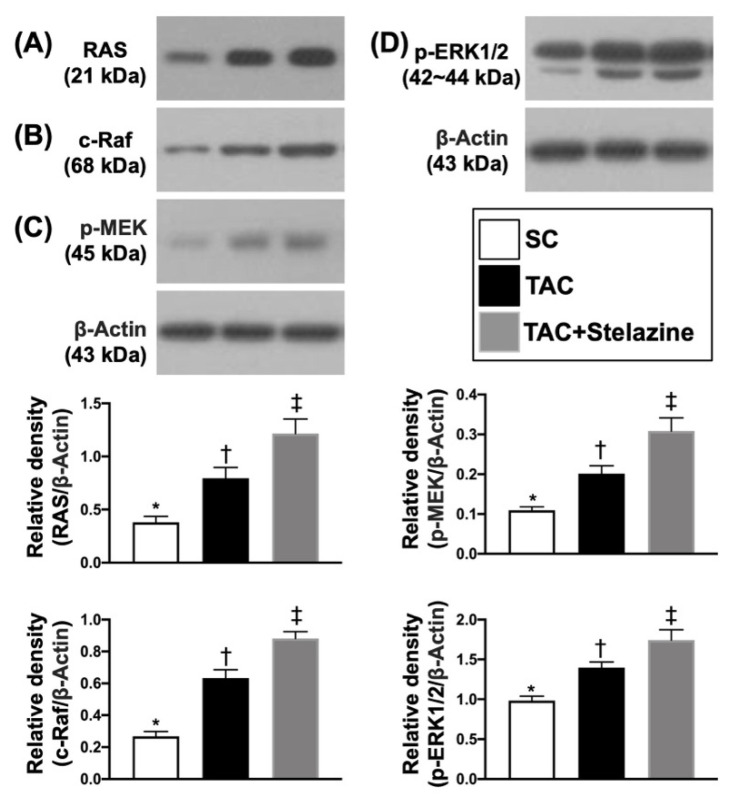
Protein expressions of EGF signalings by day 28 after TAC procedure. (**A**) Protein expression of RAS, * vs. other groups with different symbols (†, ‡), *p* < 0.0001. (**B**) Protein expression of c-RAF, * vs. other groups with different symbols (†, ‡), *p* < 0.0001. (**C**) Protein expression of p-MEK, * vs. other groups with different symbols (†, ‡), *p* < 0.0001. (**D**) Protein expression of p-ERK1/2, * vs. other groups with different symbols (†, ‡), *p* < 0.0001. All statistical analyses were performed by one-way ANOVA, followed by Bonferroni multiple comparison post-hoc test (n = 6 for each group). Symbols (*, †, ‡) indicate significance (at 0.05 level). SC = sham-operated control TAC = transverse aortic constriction; Ras = rat sarcoma; ERK = extracellular signal-regulated protein kinase; MEK = mitogen-activated protein kinase kinase; Raf = proto-oncogene c-RAF.

To further evaluate whether the cell proliferation/survival was upregulated by TCM induced by the TAC procedure, the Western blot was performed again for the harvested LV specimen. The results showed that the protein expressions of RAS, c-RAF, p-MEK, and p-ERK1/2, four indices of epidermal growth factor (EGF) signaling biomarkers, were significantly and progressively increased from groups 1 to 3, suggesting an intrinsic response to pressure-overload stress that not only related to PrP^C^-regulated signaling (i.e., PI3K/Akt/m-TOR) but also can be attributed to a rebound/feedback phenomenon in response to the cell-stress signaling blocked by Stelazine (i.e., an α-adrenergic receptor blockade) (Figure 6).

### 3.6. The Proliferation Biomarkers in LV Myocardium by Day 28 after TAC Procedure

**Figure 7:**
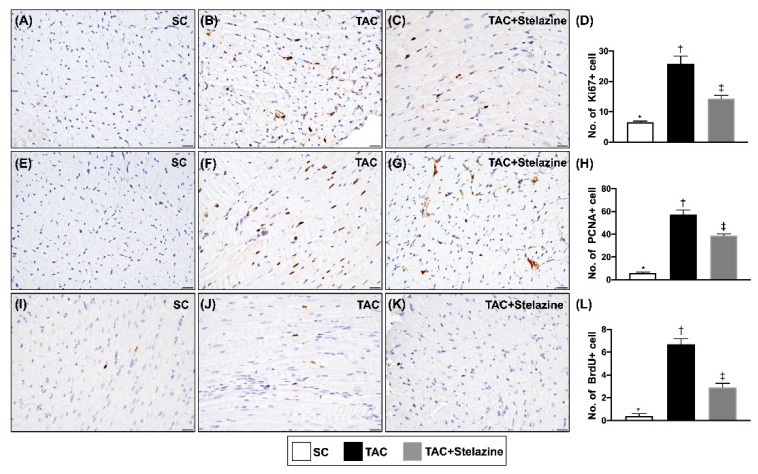
The proliferation biomarkers in LV myocardium by day 28 after TAC procedure. (**A**–**C**) Illustrating the immunohistochemical (IHC) stating (400×) for identification of Ki67+ cells (gray color). (**D**) Analytical result of number of Ki67+ cells per high-power field, * vs. other groups with different symbols (†, ‡), *p* < 0.0001. All scale bars in right lower corner represent 20 µm. (**E**–**G**) Illustrating the IHC stating (200×) for identification of PCNA+ cells (gray color). All scale bars in right lower corner represent 50 µm. (**H**) Analytical result of number of PCNA+ cells per high-power field, * vs. other groups with different symbols (†, ‡), *p* < 0.0001. (**I**–**K**) Illustrating the IHC staining (400×) for identification of BrdU+ cells (gray color). All scale bars in right lower corner represent 20 µm. (**L**) Analytical result of number of BrdU+ cells per high-power field, * vs. other groups with different symbols (†, ‡), *p* < 0.0001. All statistical analyses were performed by one-way ANOVA, followed by Bonferroni multiple comparison post-hoc test (n = 6 for each group). Symbols (*, †, ‡) indicate significance (at 0.05 level). SC = sham-operated control TAC = transverse aortic constriction; LV = left ventricular; PCNA = proliferating cell nuclear antigen; BrdU = 5-bromo-2′-deoxyuridine.

The cellular expressions of Ki67+, PCNA+ and BrdU+ cells, three indicators of cell proliferation, were significantly higher in group 2 than in groups 1 and 3, and significantly higher in group 3 than in group 1, suggesting that PrP^C^ essentially participated in regulating the cell proliferation after myocardial damage in the setting of TAC-induced TCM (Figure 7).

### 3.7. Fibrotic and Collagen-Deposition Areas in LV Myocardium by Day 28 after TAC Procedure 

**Figure 8:**
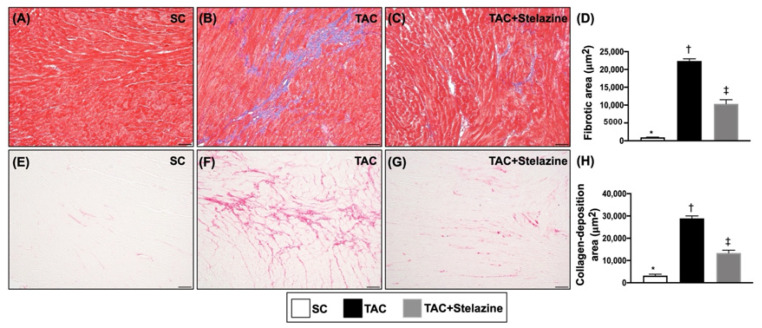
Fibrotic and collagen-deposition areas in LV myocardium by day 28 after TAC procedure. (**A**–**C**) Illustrating the microscopic finding (200×) of Masson’s trichrome staining for identification of fibrosis (blue color). (**D**) Analytical result of fibrotic area, * vs. other groups with different symbols (†, ‡), *p* < 0.0001. (**E**–**G**) Illustrating the microscopic finding (200×) of Sirius red staining for identification of collagen-deposition area (pink-red color). (**H**) Analytical result of collagen-deposition area, * vs. other groups with different symbols (†, ‡), *p* < 0.0001. All scale bars in right lower corner represent 20 µm. Symbols (*, †, ‡) indicate significance (at 0.05 level). SC = sham-operated control; TAC = transverse aortic constriction; LV = left ventricular.

To elucidate the myocardial lost and replaced by fibrosis, the IHC microscopic finding was used in the present study. The result of Masson’s trichrome stain showed that the fibrotic area was significantly higher in group 2 than in groups 1 and 3, and significantly higher in group 3 than in group 1. Additionally, the Sirius red stain showed that the collagen-deposition area exhibited an identical area of fibrosis among the three groups (Figure 8).

### 3.8. Protein Expressions of Apoptotic, Fibrotic, Pressure-Overload/Heart-Failure, and Mitochondrial/DNA-Damaged Biomarkers in LV Myocardium by Day 28 after TAC Procedure 

**Figure 9:**
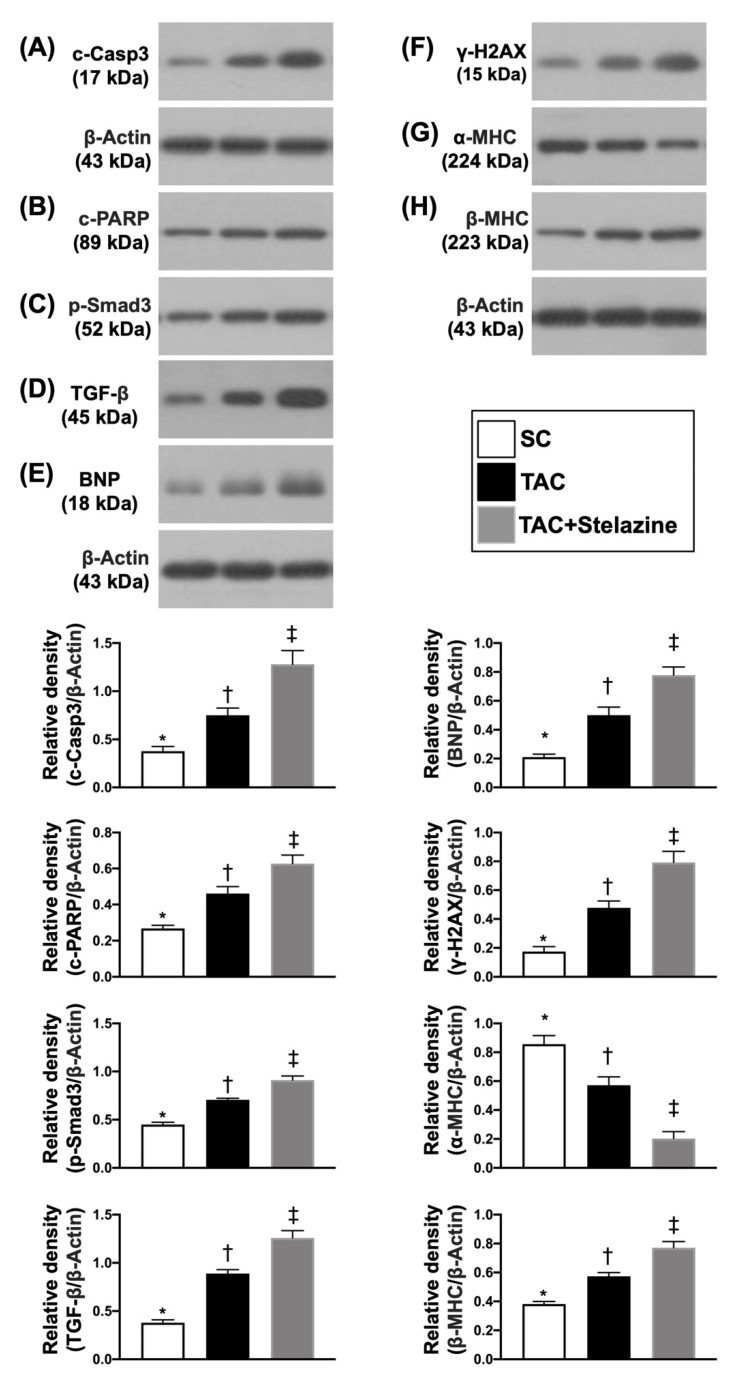
Protein expressions of apoptotic, fibrotic, pressure-overload/heart-failure, mitochondrial/DNA-damaged biomarkers in LV myocardium by day 28 after TAC procedure. (**A**) Protein expression of cleaved caspase 3 (c-Casp3), * vs. other groups with different symbols (†, ‡), *p* < 0.0001. (**B**) Protein expression of cleaved Poly (ADP-ribose) polymerase (PARP), * vs. other groups with different symbols (†, ‡), *p* < 0.0001. (**C**) Protein expression of phosphorylated (p) mothers against decapentaplegic homolog 3 (p-Smad3), * vs. other groups with different symbols (†, ‡), *p* < 0.0001. (**D**) Protein expression of transforming growth factor (TGF)-ß, * vs. other groups with different symbols (†, ‡), *p* < 0.0001. (**E**) Protein expression of brain natriuretic peptide (BNP), * vs. other groups with different symbols (†, ‡), *p* < 0.0001. (**F**) Protein expression of gamma H2A histone family member X (γ-H2AX), * vs. other groups with different symbols (†, ‡), *p* < 0.0001. (**G**) Protein expression of alpha myosin heavy chain (α-MHC), * vs. other groups with different symbols (†, ‡), *p* < 0.0001. (**H**) Protein expression of ß-MHC, * vs. other groups with different symbols (†, ‡), *p* < 0.0001. Symbols (*, †, ‡) indicate significance (at 0.05 level). SC = sham-operated control; TAC = transverse aortic constriction.

To further elucidate the protein levels of myocardial damage biomarkers in the TCM setting, the Western blot analysis was used again. As we expected, the protein expressions of cleaved caspase 3 and cleaved PARP, two indicators of apoptosis, and protein expressions of p-Smad3 and TGF-ß, two indices of fibrosis, were significantly and progressively increased from groups 1 to 3. Additionally, the protein expression of BNP, a pressure-overload/heart-failure biomarker and protein expression of beta myosin heavy chain (ß-MHC), a cardiac hypertrophy/pressure-overload biomarker, exhibited an identical pattern, whereas the protein expression of α-MHC, an indicator of reversed cardiac hypertrophy, exhibited an opposite pattern of apoptosis among the three groups (Figure 9).

## 4. Discussion

This study, which investigated the role of PrP^C^ in myocardial regeneration and recovery of left ventricular function from TAC-induced TCM, yielded several preclinically striking implications. First, at least two essential but independent signaling pathways for responding to the TCM in LV myocardium were clearly identified. Second, a rebound/feedback phenomenon, i.e., more markedly upregulated cell proliferation/survival biomarkers (RAS, c-RAF, p- MEK, and p-ERK1/2), due to the cell-stress signaling blocked by Stelazine (i.e., an α-adrenergic receptor blockade) was distinctively observed in the in vivo study. Third, Stelazine therapy was surprisingly recognized to improve LV function in the setting of TAC-induced TCM.

An important finding in the in vitro study was that the protein expression of PrP^C^ in H9C2 cells was markedly suppressed by isoproterenol and H_2_O_2_ treatment, suggesting that this gene is vulnerable to ß-adrenergic agonist and oxidative stress damage. Interestingly, the protein expressions of cell-stress signaling biomarkers (i.e., PI3K/Akt/m-TOR) were found to be consistently downregulated as the PrP^C^ in the same therapeutic conditions. On the other hand, when looking at the situation of PrP^C^ gene overexpression in H9C2 cells, we identified that not only the PI3K/Akt/m-TOR signaling but also the RAS/c-RAF/p-MEK1/2//p-ERK1/2 signaling were substantially upregulated, whereas the Stelazine (a PrP^C^ inhibitor) treatment significantly downregulated and siRNA-PrP^C^ further significantly downregulated the protein expressions of these two signaling pathways. Interestingly, recent studies demonstrated that PI3K/Akt/m-TOR [34,35] and MAPK family signaling cascades [36] were markedly upregulated in response to ischemic stimulation. In this way, our findings, in addition to corroborating with the findings of these studies [34,35,36], implied that these two cell-stress/cell-proliferation signalings were dependently regulated by PrP^C^ expression, at least in the condition of cell culturing.

Interestingly, when we carefully looked at the time courses of LVEF, we found that the LVEF did not differ among groups 2 (i.e., TCM) and 3 (TCM + Stelazine/administered from day 7 after TCM induction) by the time interval of 7 days after TCM induction. However, when we inspected the LVEF at the time points of days 14, 21, and 28 after TCM induction, this parameter was significantly higher in group 3 than in group 2. Indeed, we remain uncertain regarding why the PrP^C^ upregulated cell-stress signaling (PI3K/Akt/m-TOR) was already inhibited by Stelazine. Instead of worsening, the LVEF became furthermore improved. Perhaps, it could be attributed to the afterload reduction in Stelazine therapy, especially in the designated setting to TCM.

It is well-known that the RAS/c-RAF/MEK1/2//ERK1/2 is a crucial cell-proliferation and cell-survival signaling [37,38]. Additionally, it is also well recognized that PI3K/Akt/m-TOR is a principal cell-stress/cell-survival/cell death signaling [39,40]. A principal finding in the in vitro study was that overexpression of PrP^C^ not only upregulated the cell-stress signaling pathway (i.e., PI3K/Akt/m-TOR) but also augmented the cell-proliferation and cell-survival signaling (RAS/c-RAF/MEK1/2//ERK1/2), whereas silencing of PrP^C^ (i.e., siRNA-PrP^C^) gene expression substantially downregulated these signaling parameters, suggesting that PrP^C^ could play a crucial role on regulating these two signaling pathways in response to cells encountering harsh environments.

**Figure 10:**
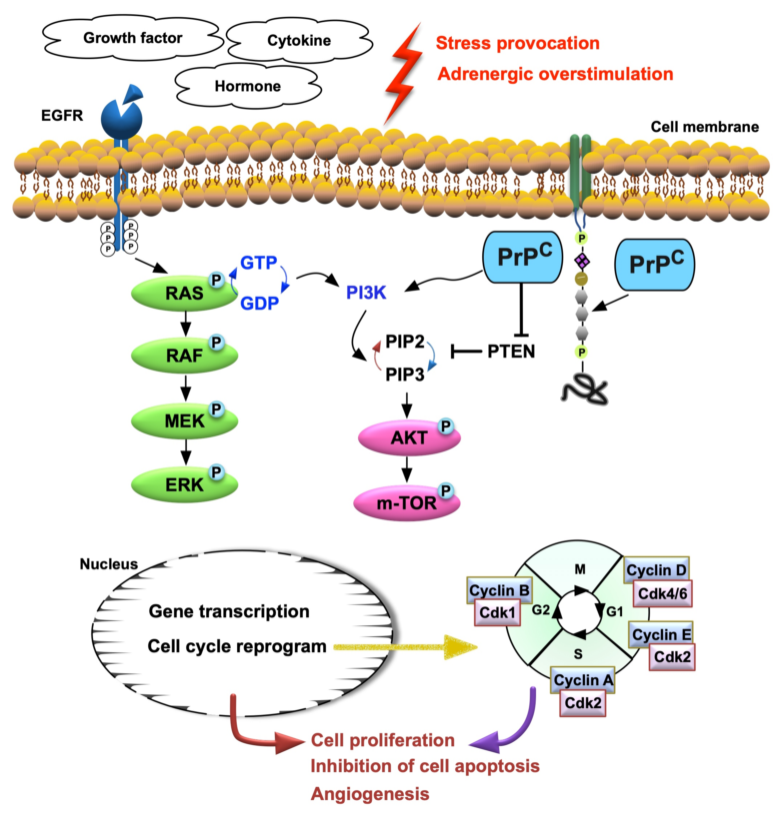
Schematically illustrating the proposed mechanism of PrP^C^ involving the regeneration of myocardial tissues after apical ballooning. PrP^C^ = cellular prion protein; EGFR = epidermic growth factor.

Intriguingly, when viewing the result from the in vivo study, we also found that the role of PrP^C^ on regulating the cell-stress signaling pathway (i.e., PI3K/Akt/m-TOR) and cell cycle (i.e., cyclin E and D) was comparable with the finding from the in vitro study and the P-TEN reasonably displayed an opposite pattern of cell-stress signaling pathway among the three groups. On the other hand, the protein expressions of cell-survival signaling (RAS/c-RAF/MEK1/2//ERK1/2) were identified to significantly and progressively increase from groups 1 to 3 (i.e., from SC, followed by TCM only, finally by TCM + Stelazine), suggesting in vivo result was not comparable with that of the in vitro and could be due to the cell-stress signaling blocked by Stelazine (i.e., an α-adrenergic receptor blockade), resulting in a rebound/feedback phenomenon. Additionally, the cellular levels of cell proliferative biomarkers (i.e., cellular uptake of BrdU, PCNA+ cells, and Ki67+ cells) were lowest in group 1 but surprisingly higher in group 2 than in group 3. We suggested that these discrepant findings between in vitro and in vivo studies could be, at least in part, due to the RAS/c-RAF/MEK1/2//ERK1/2 signaling was not affected by PrP^C^ but affected by an intrinsic response to ischemic stimulation + post-day 14 relieving the slipknot in the in vivo environment (refer to Figure 10). On the other hand, the cell proliferation (i.e., cellular uptake of BrdU, PCNA+ cells, and Ki67+ cells) could be dominantly regulated by PrP^C^ and PI3K/Akt/m-TOR as well as by post-day 14 relieving the slipknot rather than by the RAS/c-RAF/MEK1/2//ERK1/2 signaling in the in vivo environment (refer to Figure 10).

Our previous studies [30,32,33] have clearly identified that molecular-cellular perturbations, including those of inflammation, apoptosis, oxidative stress, fibrosis, and DNA-damaged biomarkers, were substantially released in the setting of ischemia. In the present studies, the protein expressions of apoptosis (i.e., cleaved caspase 3 and cleaved PARP), fibrosis (p-Smad3 and TGF-ß), DNA-damage marker (γ-H2AX) and pressure-overload/heart failure biomarkers (ß-MHC/BNP), as well as the cellular levels of fibrotic/collagen-deposition areas in LV myocardium, were significantly and progressively increased from groups 1 to 3. Our findings, therefore, were comparable with the findings of our previous studies [30,32,33]. Intriguingly, the findings of these biomarkers from the in vivo study were consistent in every respect and, therefore, strongly demonstrated why the LVEF was greatly improved in TAC + Stelazine group after relieving the tie in the transverse aortic arch than in the TAC group only. These findings once again explained mainly the key factor can be due to reduction in the afterload by Stelazine therapy rather than upregulation of PrP^C^ in the myocardium.

Overwhelming evidence links the NLRP3 inflammasome and the IL-1 cytokines with the pathogenesis of cardiovascular diseases, nonischemic injury to the myocardium, and worsening heart failure [41]. Intriguingly, canakinumab and anakinra, two IL-1β antagonists, have been established to effectively prevent the recurrence of ischemic events in patients with prior acute myocardial infarction [41]. Thus, the targeted inhibitors to block the IL-1 isoforms and possibly oral NLRP3 inflammasome inhibitors may potentially target a wide spectrum of cardiovascular diseases [41]. Furthermore, abundant data have shown that adrenergic cardiac innervation dysfunction plays a crucial role in TCM patients. Interestingly, a previous randomized control trial has shown that α-lipoic acid (ALA) treatment, a drug for improving the adrenergic cardiac innervation, could improve the adrenergic cardiac innervation in TCM patients [42], implicating a therapeutic treatment to target the heart sympathetic dysfunction could be of fundamental importance in the future.

## 5. Study Limitation

This study has limitations. First, the study period was only 28 days that might not provide enough time for observing the natural recovery of heart function in TCM (i.e., it may need much longer time to recover.), especially when we took the PrP^C^ as a potential role of tissue regeneration and improvement of the heart function into consideration. Second, the use of the Stelazine dosage in mice was only based on the previous report [31] without a stepwise increase in the concentration. Accordingly, the result of this study did not provide adequate information regarding the optimal dosage of Stelazine on improving the LVEF. Third, this study did not measure the circulating level of catecholamines, i.e., the markers for different degrees of cardiac denervation and the recovery of left ventricular function. This could be one paramount importance of diagnostic markers for apical ballooning. Finally, although extensive works had been performed in the present study, the underlying mechanism for PrP^C^ on regulating myocardial regeneration might not be fully delineated. Accordingly, we proposed the mechanisms underlying the role of PrP^C^ on regulating the cell-stress/cell-proliferative signalings for myocardial regeneration in the setting of apical ballooning syndrome merely based on the results of our study (Figure 10).

In conclusion, cell-stress/cell-proliferation signalings were activated by upregulation of PrP^C^ as a consequence of LV intrinsic response to pressure overload as well as myocardial regeneration. However, the result of upregulation of PrP^C^ was inferior to extrinsic Stelazine therapy for improving the LVEF in the setting of a shorter interval of TCM in mice.

## Data Availability

All generated and analyzed data used to support the findings of this study are included within the article.
